# Findings of a videofluoroscopic swallowing study in patients with dysphagia

**DOI:** 10.3389/fneur.2023.1213491

**Published:** 2023-08-04

**Authors:** Qingjia Cui, Bing Wei, Yuan He, Qian Zhang, Weiwei Jia, Haiying Wang, Jianing Xi, Xin Dai

**Affiliations:** ^1^Rehabilitation Centre of Otolaryngology-Head and Neck, Beijing Rehabilitation Hospital, Capital Medical University, Beijing, China; ^2^Speech Rehabilitation Department of Neurorehabilitation Center, Beijing Rehabilitation Hospital, Capital Medical University, Beijing, China; ^3^Beijing Rehabilitation Hospital, Capital Medical University, Beijing, China

**Keywords:** dysphagia, videofluoroscopic swallowing study, qualitative analysis, quantitative analysis, parameters

## Abstract

**Objective:**

Swallowing examination is crucial in patients with dysphagia. We aimed to compare qualitative and quantitative videofluoroscopic swallowing study (VFSS) results to provide reference for standardizing quantitative parameters.

**Materials and methods:**

In total, 117 patients with dysphagia were included, 38 with Parkinson’s disease and 39 and 40 in convalescence following cerebral hemorrhage and infarction. VFSS was both qualitatively and quantitatively analyzed.

**Results:**

A significant difference of Oral transit time was found between the oral motor function grades (*p* < 0.001), also was swallowing reaction times found between swallowing reaction duration grades (*p* < 0.001), and soft palate lift duration between the soft palate lift grades (*p* < 0.001). Superior hyoid bone movement (*p* < 0.001), anterior hyoid bone movement (*p* < 0.001), hyoid pause time (*p* < 0.001), and hyoid movement duration (*p* = 0.032) had significant differences between the hyoid laryngeal complex movement grades, as did the pharyngeal cavity transit time among the cricopharyngeal muscle opening duration grades (*p* < 0.001). The laryngeal vestibule closure duration differed among the glottic closure grades (*p* < 0.001). No statistically significant difference in upper esophageal sphincter opening diameter (*p* = 0.682) or duration (*p* = 0.682) among the cyclopharyngeal muscle opening duration grades. The pharyngeal area at rest did not significantly differ among the different vallecular residue (*p* = 0.202) and pyriform sinus residue (*p* = 0.116) grades.

**Conclusion:**

Several quantitative parameters can reflect the swallowing assessment process well. Further optimization of quantitative parameters is recommended.

## 1. Introduction

Dysphagia is a term that describes abnormal swallowing function caused by various factors in different parts of the body, with a prevalence ranging from 19% ~ 81% (1). The incidence of dysphagia caused by stroke in the acute phase was shown to be approximately 46.3%; during the period of convalescence, however, the incidence increased to 56.9 ~ 81.0% (2). The incidence rate of dysphagia varies in those with progressive neurological conditions. In patients with Parkinson’s disease (PD), the incidence rate is approximately 50 ~ 87.1%; however, the risk of dysphagia in elderly patients with PD is about twice that in young patients, and the risk of dysphagia in patients with PD with a higher Hoehn and Yahr stage is about three times that in patients with a lower stage (3, 4). The main clinical manifestations of dysphagia include weakening of muscles involved in chewing, leading to food remaining in the mouth or leaking out of the mouth after swallowing, cough, reflux, and aspiration after swallowing (5). Related complications also include the consequences of aspiration (such as pneumonia, repeated cough, and asphyxia) and changes in diet and fluid intake (such as malnutrition, dehydration, decreased quality of life, and social isolation) (1). A literature review and analysis revealed that a series of complications caused by dysphagia irreversibly affected the physical and mental health of patients and their families to a certain extent (6). Ensuring that patients with dysphagia receive timely and correct examination and undergo appropriate and effective treatment and rehabilitation can promote functional recovery and reduce the occurrence of complications (7).

A videofluoroscopic swallowing study (VFSS) is a special type of examination involving X-ray fluoroscopy that is used to assess the movement of components involved in swallowing, including the mouth, pharynx, larynx, and esophagus. Analysis of VFSS data can lead to the discovery of swallowing function abnormalities from spot film and video, including further frame-by-frame and slow playback analysis. This examination is a widely used and relatively mature technique for assessing the swallowing function of patients in clinical practice, as it can directly reflect dynamic changes in the functions of the participating organs. VFSS is mainly used to assist in the diagnosis of swallowing disorders and is considered to be the “ideal method” for swallowing disorders examination, and it remains the “gold standard” for diagnosis (8).

The qualitative analysis of VFSS data mainly involves the assessment of the presence of aspiration and the evaluation of tongue movement, cricopharyngeal muscle function, swallowing reflex, laryngeal lift, epiglottic vallecula, and/or pyriform fossa retention via angiography to determine the swallowing function of patients (9, 10). Currently, qualitative analysis is the most widely used method for clinical evaluation, owing to its simplicity and high efficiency. However, there are certain shortcomings related to qualitative analyses. For example, the comprehensiveness and accuracy of the evaluation content are dependent on the patient’s cooperation during and the methodology of the imaging examination, the quality of the recorded video, the technical expertise of the imaging personnel, and the analytical aptitude and experience of the rehabilitation physician or therapist evaluating the findings. Therefore, qualitative assessments fail to satisfy the criteria required for objective clinical evaluations and scientific research.

With the progressive advancements in imaging technology, a dynamic swallowing method has been developed to analyze the VFSS in recent 30 years (11, 12). The scholars have attempted to conduct quantitative VFSS analyses by recording the imaging data using a digital acquisition system at a speed of 30 frames/s, browsing it frame-by-frame, and quantifying the temporal and kinematic parameters involved in the swallowing process (13, 14). Further standardization and validation is required before such methods can be widely used in quantitative clinical evaluations and scientific research involving patients with dysphagia. Therefore, this study aimed to conduct a comparative analysis of quantitative and qualitative VFSS findings among patients with clinical dysphagia to obtain more valuable information and then use analysis software to quantify and automatically analyze the swallowing function of patients and apply it to clinical practice.

## 2. Materials and methods

### 2.1. Study design and participants

The participants were selected from patients with dysphagia who were treated in the Speech Rehabilitation Department of Beijing Rehabilitation Hospital affiliated with Capital Medical University from June 2022 to December 2022. The inclusion criteria were as follows: (1) patients who experienced cerebral hemorrhage and cerebral infarction who met the relevant diagnostic criteria formulated by the Fourth National Conference on Cerebrovascular Diseases (1995) and received a definitive diagnosis of stroke based on head magnetic resonance imaging (MRI); (2) patients with PD who met the MDS clinical diagnostic criteria for Parkinson’s disease (15); (3) patients with varying degrees of comorbid dysphagia based on Expert consensus on evaluation and treatment of swallowing disorders in China (2017); (4) those with stable vital signs who were able to pay good attention to their surroundings, exhibited no serious cognitive impairment, and were able to cooperate to complete the required angiography. The exclusion criteria were as follows: (1) patients with complete or severe dysphagia; (2) those with an allergy to the contrast agent; (3) those with dysfunction of the heart, kidney and other organs; (4) patients with organic lesions of the esophagus, pharynx, and mouth; (5) those with a recent history of treatment with muscle relaxants and sedatives that could affect swallowing function; (6) individuals with cognitive impairment and mental illness; and (7) those with thyroid disease, local infection, other local diseases of the throat, or chronic respiratory diseases.

### 2.2. Sample size

We calculated the required sample size based on previous research of the incidence of stroke in the acute phase, stroke in convalescence and Parkinson’s disease. Based on an alpha level of 0.05 and a power of 0.90, we estimated that we would require a total of 60 subjects, divided among three groups.

### 2.3. Videofluoroscopic swallowing study methodology

All patients underwent VFSS examination with an OPERA digital multifunctional gastrointestinal imaging device (GMM Group). Images were jointly collected by an radiologist with many years of diagnostic experience and an experienced rehabilitation physician with experience treating dysphagia. Each patient swallowed a 5 mL volume and medium consistency of food paste, containing a thickener and an iodohydrin contrast agent. We believe that this choice is appropriate in terms of safety, with a moderate degree of risk of aspiration, meanwhile it can also cause a series of swallowing movements, the quantitative data can be collected well, which basically meets the needs of this study. The state of the food was recorded as it passed through the mouth, pharynx, and esophagus in the anteroposterior and lateral orientations through video fluoroscopy. In VFSS, the swallow was performed once.

### 2.4. Qualitative analysis methods

According to the VFSS, abnormal swallowing function was assessed and the related physiological and pathological components were analyzed, including the oral motor function, swallowing reaction function, soft palate lift function, hyoid–laryngeal complex movement, cricopharyngeal muscle opening duration, glottic closure, and the presence of vallecular and pyriform sinus residues. A double-blind evaluation of the VFSS findings was conducted by a PhD candidate studying speech rehabilitation and a professional physician with many years of experience in diagnosing dysphagia. The two doctors independently completed the evaluation. Any discrepancies between the two researchers were reconciled by discussion between these two individuals before determining the final results. Eight qualitative components related to swallowing function were graded (16) and scored as listed in Table 1.

**Table 1:** Grades and scoring of the qualitative items.

Item	Grade	Score
Oral motor	Normal	3
	Impaired	2
	Severe impaired	1
Swallowing reaction	Normal	2
	Delayed	1
Soft palate lift function	Normal	3
	Impaired	2
	Severe impaired	1
Hyoid laryngeal complex movement	Intact	3
	Inadequate	2
	None	1
Cricopharyngeal muscle opening duration	Normal	3
	Delayed	2
	Severe delayed	1
Glottic closure	Intact	3
	Inadequate	2
	None	1
	Unable to cooperate	0
Vallecular residue	None	3
	<50%	2
	> 50%	1
Pyriform sinus residue	None	3
	<50%	2
	>50%	1

### 2.5. Quantitative analysis methods

The temporal and kinematic parameters of the VFSS were collected and recorded in a double-blind manner by a speech rehabilitation physician in the field of dysphagia and a speech rehabilitation therapist with extensive experience in the treatment of dysphagia. The average of each value determined by the two evaluators was used as the final result. If the two values differed, they were discussed, and the final result was determined through negotiation.

**Figure 1:**
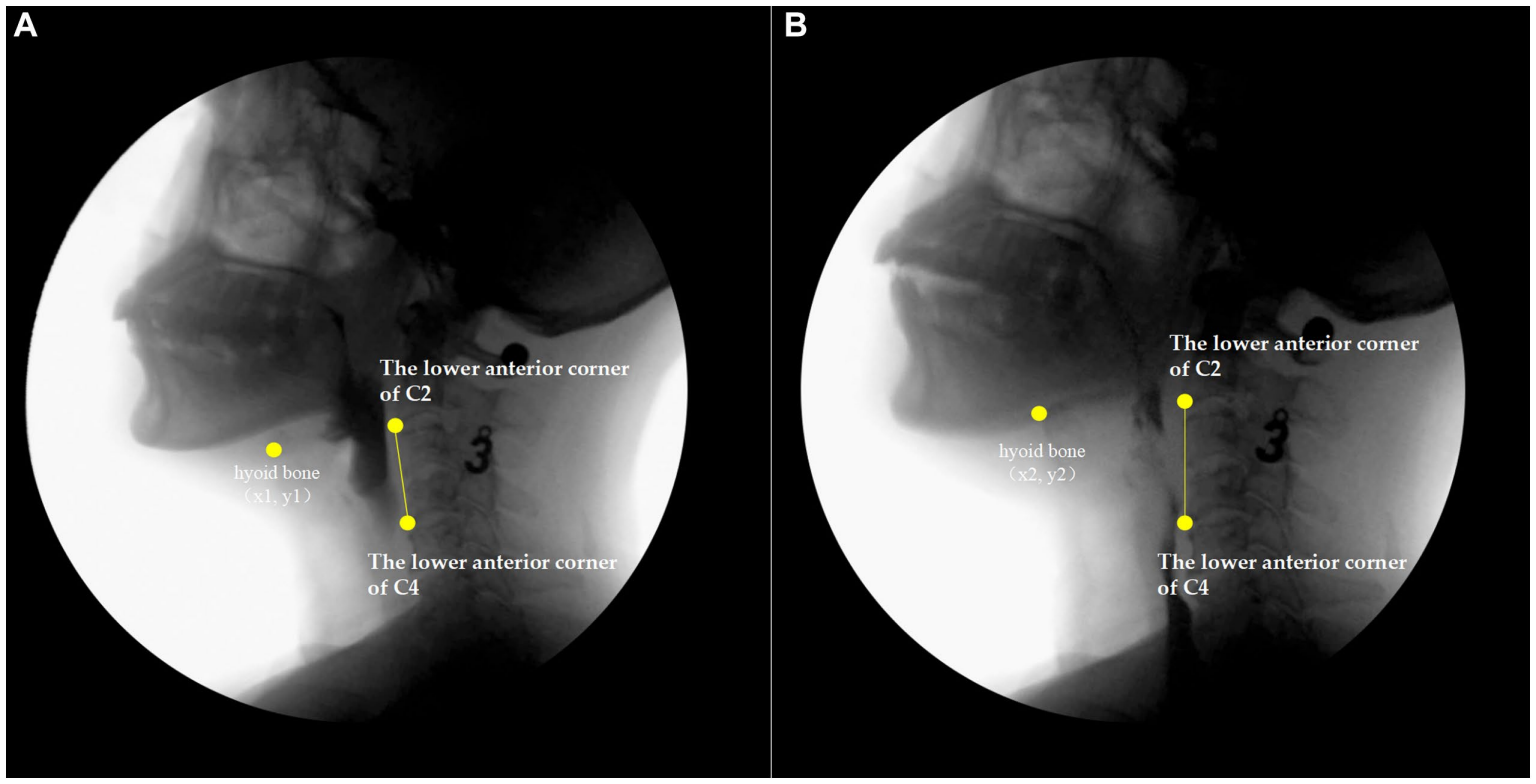
Hyoid bone superior and anterior movement based on videofluroscopy. **(A)** screenshot showing the initial state of the hyoid bone after rotation; **(B)** screenshot of the time point at which the hyoid bone has been lifted to the highest and farthest position after rotation. x1 is the horizontal coordinate of the hyoid resting position, and y1 is the vertical coordinate of the hyoid resting position; x2 is the abscissa of the farthest point of hyoid motion, and y2 is the ordinate of the farthest point of hyoid motion; C4xl is the abscissa of the lower anterior corner of C4 in the hyoid resting position, and C4yl is the ordinate of the lower anterior corner of C4 in the hyoid resting position; C4x2 is the abscissa of the lower anterior corner of C4, the farthest point of hyoid motion, and C4y2 is the ordinate of the lower anterior corner of C4, the farthest point of hyoid motion.

An 8 mm reference ball was set up as calibration index for calculating distances in the analysis of kinematic parameters. Quantitative VFSS analysis contains four kinematic parameters and eight temporal parameters. The specific kinematic parameters were assessed including the following: (1) hyoid bone superior movement (HSM), the vertical distance between the lowest and highest positions of the hyoid bone during the swallowing process, from the movement of the hyoid bone to its return to its original position and (2) hyoid bone anterior movement (HAM), the horizontal distance between the lowest and highest positions of the hyoid bone (17, 18). The interception method is shown in Figures 1A,B. The line between the lower anterior corners of C2 and C4 represented the vertical axis, which was made neutral by rotation and lied perpendicular to the horizontal axis of the image, C2 and C4 vertebrae shown in Figures 1A,B, then sent their coordinates (x1, y1), (x2, y2), (C4x1, C4y1), (C4x2, C4y2) to the analysis software, and the hyoid bone movement was calculated using the built-in Equations (1) and (2).

**Figure 2:**
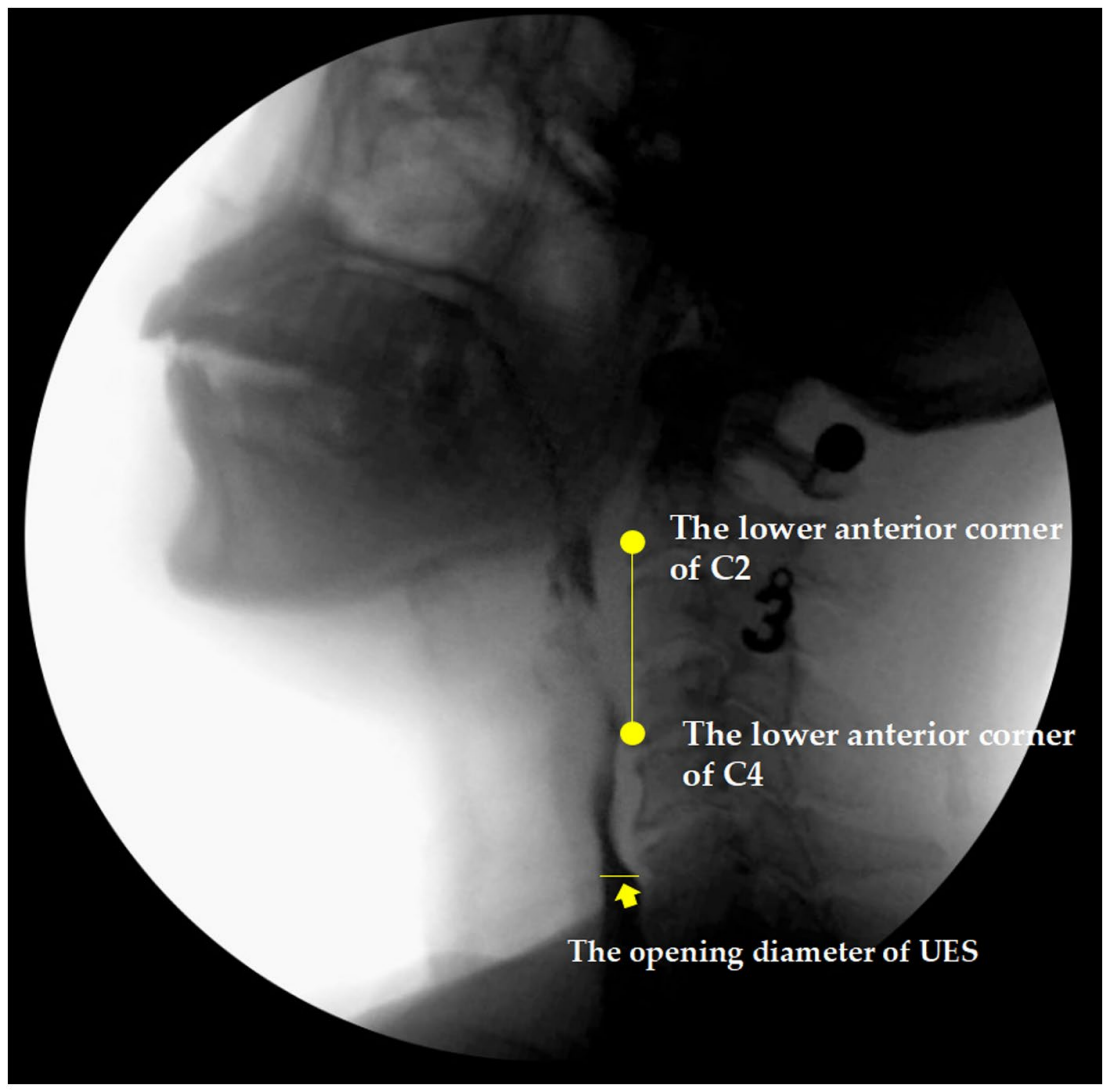
The upper esophageal sphincter (UES) based on videofluroscopy.

(1) HAM=(x2x1)(C4x2C4x1),

(2) HSM=(y2y1)(C4y2C4y1),

The kinematic parameters also included: (3) the upper esophageal sphincter (UES) opening diameter, as shown in Figure 2, the width of the narrowest part of the pharyngoesophageal sphincter in the lateral image at the maximum degree of expansion induced by the mass in a single swallow, with the measurement line perpendicular to the edge of the spine (19); and (4) the pharyngeal area at rest, the minimum lateral area of the swallowed mass and pharyngeal cavity upon contraction in one mouthful (20).

The eight time parameters assessed have been included as follows: (1) the oral transit time, defined as the time interval between the food completely entering the mouth and being pushed by the tongue muscle resulting in a change in shape until the time at which the head of the food ball reached the intersection between the mandibular branch and the base of the tongue (21); (2) the soft palate elevation time, the interval between the time when initial soft palate contact and the time at which the posterior pharyngeal wall moves down to its original position (22); (3) the hyoid at rest duration, the length of time when the hyoid bone is completely at rest position during swallowing (22); (4) the hyoid movement duration, defined as the time interval between the initiation of the forward and downward movement of the hyoid bone and the return of the hyoid bone to its resting position (18); (5) the UES opening duration, representing the time interval between the beginning of the opening and the complete closing after the mass reaches the UES (14); (6) the swallow reaction time, which is the time interval between the head of the mass reaching the intersection of the lingual and mandibular branches and the start of the swallowing phase, marked by the initiation of hyoid bone movement (14); (7) the pharyngeal transit time, defined as the time interval between the head of the food mass passing the intersection of the lingual and mandibular branches and the tail of the food mass passing the UES (23); and (8) the laryngeal vestibule closure (LVC) duration, representing the time interval between the closing and reopening of the laryngeal vestibule (23).

### 2.6. Statistical analysis

Statistical Package for the Social Sciences (SPSS) version 22.0 software was used for statistical analysis. One-way ANOVA and Bonferroni post-hoc tests were used to assess the differences in the mean age or the mean score of the clinical groups. Kruskal–Wallis test was used for comparison among multiple groups. *p* < 0.05 is statistically significant. The measurement data conforming to a normal distribution is expressed as mean ± standard deviation, and the measurement data not conforming to a normal distribution is expressed as median (25th percentile–75th percentile).

To evaluate different dimensions of the qualitative values, principal component analysis (PCA) was performed. PCA is concerned with establishing which linear components exist within the data and how a particular variable might contribute to that component. The PCA was conducted with all 12 items into analysis to maximize the loadings of the variables onto one factor (the factor that intersects the cluster) and minimize them on the remaining factor(s). The assumptions were fulfilled (Bartlett’s test was highly significant and the Kaiser–Meyer–Olkin (KMO) measure of sampling adequacy > 0.6). The analysis of the association between the qualitative results and quantitative results was performed using Pearson’s correlation coefficients.

## 3. Results

### 3.1. Overview

A total of 151 patients with dysphagia were considered for inclusion, 123 of whom agreed to participate. Of these 123 patients, three did not meet the inclusion criteria (one case had a respiratory infection and two cases experienced a recurrence of sudden cerebral infarction) and three temporarily refused to undergo VFSS. Ultimately, 117 patients with dysphagia completed the study and were included in the analysis. Table 2 summarizes the detailed demographic data of the participants and the cause of dysphagia.

**Table 2:** The demographic characteristics of the patients with dysphagia.

Variables	Patients
Sample size	117
Sex (male/female)	61/56
Mean age (years)	61.31 ± 12.38 (27–83)
Type	
Parkinson’s disease	38
Cerebral hemorrhage	39
Cerebral infarction	40

### 3.2. Qualitative grading and quantitative value matching of VFSS

The mean and standard deviation values of the quantitative items according to the different grades of the qualitative items of the VFSS are listed in Table 3. Among these items, the oral transit time significant differed among the different grades of qualitative oral motor function; with poorer oral motor function resulting in significant prolongation of the oral transit time. The quantitative swallowing reaction time significantly differed among the qualitative swallowing reaction grades; that is, the worse the swallowing reaction grade, the longer the swallowing reaction time. Similarly, as the grade of the soft palate lift decreased, the soft palate lift duration was significantly prolonged. Weakening of the hyoid–laryngeal complex movement resulted in significant shortening of the HSM and HAM and prolongation of the hyoid pause time and hyoid movement duration. Poorer cricopharyngeal muscle opening durations resulted in significant prolongation of the pharyngeal cavity transit time, poorer glottis closure grades resulted in significant prolongation of the LVC duration.

**Table 3:** Qualitative grading and quantitative value matching of videofluoroscopic swallowing study (VFSS).

Qualitative items	Grade	*n*	Quantitative items	*F*	*p*
			Oral transit time (s)		
Oral motor	Normal	59	2.75 ± 1.95	52.103	<0.001
	Impaired	34	5.40 ± 3.41		
	Severe impaired	24	11.46 ± 5.94		
			Swallowing reaction time (s)		
Swallow reaction	Normal	44	1.00 ± 0.99	4.207	<0.001
	Delayed	72	0.55 ± 0.54		
			Soft palate elevation time (s)		
Soft palate lift	Normal	81	1.99 ± 1.17	34.508	<0.001
	Impaired	20	2.17 ± 1.43		
	Severe impaired	16	8.24 ± 6.99		
			HSM (mm)		
Hyoid laryngeal complex movement	Intact	24	10.55 ± 7.60	9.058	<0.001
	Inadequate	51	7.97 ± 6.52		
	None	42	4.41 ± 3.45		
			HAM (mm)		
	Intact	24	9.28 ± 5.59	9.801	<0.001
	Inadequate	51	14.45 ± 7.10		
	None	42	16.07 ± 4.88		
			Hyoid at rest time (s)		
	Intact	24	0.11 ± 0.07	7.479	0.001
	Inadequate	51	0.08 ± 0.06		
	None	42	0.06 ± 0.03		
			Hyoid movement duration (s)		
	Intact	24	1.72 ± 0.50	3.551	0.032
	Inadequate	51	2.14 ± 1.11		
	None	42	2.59 ± 1.76		
			UES opening diameter (mm)		
Cricopharyngeal muscle opening duration	Normal	58	6.93 ± 2.34	0.384	0.682
	Delayed	35	6.67 ± 1.56		
	Severe delayed	24	6.56 ± 1.85		
			UES opening duration (s)		
	Normal	58	0.90 ± 0.25	1.823	0.166
	Delayed	35	0.90 ± 0.34		
	Severe delayed	24	0.78 ± 0.27		
			Pharyngeal cavity transit time (s)		
	Normal	58	1.54 ± 0.67	15.540	<0.001
	Delayed	35	1.64 ± 0.86		
	Severe delayed	24	2.89 ± 1.68		
			LVC duration (s)		
Glottic closure	Intact	68	0.71 ± 0.38	8.343	<0.001
	Inadequate	22	1.07 ± 0.89		
	None	12	1.44 ± 1.05		
	Unable to cooperate	15	/		
			Pharyngeal area at rest (%)		
Vallecular residue	None	5	36.60 ± 17.39	1.619	0.202
	<50%	60	44.13 ± 30.16		
	>50%	53	36.38 ± 12.19		
			Pharyngeal area at rest (%)		
Pyriform sinus residue	None	19	41.75 ± 15.39	0.116	0.890
	<50%	48	41.44 ± 27.14		
	> 50%	50	38.90 ± 18.97		

VFSS: video fluoroscopic swallowing study; HSM: hyoid bone superior movement; HAM: hyoid bone anterior movement; UES: upper esophageal sphincter; LVC: laryngeal vestibule closure.

The UES opening diameter and duration both decreased as the grade of the cricopharyngeal muscle opening duration increased, although neither parameter significantly differed between the different qualitative grades of cricopharyngeal muscle opening. There was no statistically significant difference in the pharyngeal cavity contraction rate among the different qualitative grades of the presence of vallecular and pyriform sinus residues. Almost all patients with dysphagia exhibited vallecular residues (96.58%); of them, 53.10% had a small amount, whereas 46.90% had a large amount. No vallecular residue was observed in 4.27% of participants. Approximately 4/5 patients with dysphagia exhibited pyriform sinus residues (83.76%); of them, 48.98% had a small amount and 51.02% had a large amount.

### 3.3. The qualitative total value of VFSS corresponds to the quantitative value

The mean and standard deviation values of the quantitative kinematic parameters for the different qualitative total value categories are listed in Table 4. Among them, the HSM, HAM, and pharyngeal area at rest significantly differed according to the different qualitative total value classifications. However, the opening diameter of the UES did not significantly differ between the qualitative total value classifications, although the range decreased as the qualitative total value classification worsened.

**Table 4:** Comparison to quantitative values of motion parameters (mm) by qualitative total value.

Qualitative total value	*n*	HSM	HAM	UES opening diameter	Pharyngeal area at rest (%)
Good (24′–20′)	29	4.83 ± 3.98	10.17 ± 6.33	7.20 ± 1.38	41.80 ± 18.51
Passable (19′–13′)	68	8.50 ± 7.74	14.83 ± 6.59	6.70 ± 1.90	41.35 ± 24.74
Poor (12′–8′)	20	8.87 ± 3.65	16.31 ± 4.52	6.20 ± 1.04	55.00 ± 9.54
***F***		3.745	7.438	1.225	3.304
***p***		0.027	<0.001	0.230	0.040

HSM: hyoid bone superior movement; HAM: hyoid bone anterior movement; UES: upper esophageal sphincter.

For the time parameters, the mean and standard deviation values of the quantitative measures for each qualitative total value range are listed in Table 5. Every quantitative measure of the time parameters significantly differed according to the qualitative total value classification. As the qualitative total grading worsened, the oral transit time, soft palate lift duration, hyoid pause time, hyoid movement duration, swallowing reaction time, pharyngeal cavity transit time, and LVC duration were significantly prolonged. The opening duration of the UES was not significantly different between the groups with good and poor qualitative values; however, it was significantly longer than that in the group with poor results.

**Table 5:** Comparison of quantitative values of time parameters by qualitative total value.

Qualitative total value	*n*	Oral transit time (s)	Soft palate lift duration (s)	Hyoid at rest duration (s)	Hyoid movement duration (s)	UES opening duration (s)	Swallow reaction time (s)	Pharyngeal cavity transit time (s)	LVC duration (s)
Good (24′–20′)	29	3.20 ± 3.14	1.72 ± 1.42	0.08 ± 0.06	1.72 ± 0.55	0.91 ± 0.31	0.52 ± 0.43	1.44 ± 0.51	0.59 ± 0.11
Passable (19′–13′)	68	3.93 ± 3.34	2.52 ± 2.20	0.08 ± 0.06	2.14 ± 1.11	0.92 ± 0.29	0.65 ± 0.57	2.01 ± 1.00	0.92 ± 0.78
Poor (12′–8′)	20	11.3 ± 10.0	8.98 ± 8.60	0.05 ± 0.02	3.20 ± 2.16	0.70 ± 0.17	2.24 ± 2.06	2.75 ± 2.22	1.02 ± 0.36
***F***		18.956	24.181	3.696	8.493	5.230	23.272	6.880	3.645
***p***		<0.001	<0.001	0.028	<0.001	0.007	<0.001	0.002	0.029

UES: upper esophageal sphincter; LVC: laryngeal vestibule closure.

No., number.

### 3.4. The correlation between the quantitative total value and qualitative total value

Bartlett’s test of sphericity was highly significant (*p* < 0.001), indicating that correlations between the items were sufficiently high for PCA analysis. The KMO measure verified the sampling adequacy for the analysis, and KMO resulted in a value of 0.604, which is in agreement with the recommended assumptions. An initial analysis was run to obtain eigenvalues for each component in the data. Five components had eigenvalues over Kaiser’s criterion of 1 and this combined explains 69% of the variance. Then the factor expression of quantitative total value is obtained, and the quantitative total value is calculated. The correlation between the qualitative total value and the quantitative total value was statistically significant (*p* = 0.036 < 0.05), the correlation coefficient was 0.194, which shows that there is no relevance between them.

## 4. Discussion

The swallowing process involves a series of complex, highly coordinated, and fixed muscle movement behaviors. Applying instrument to evaluate swallowing can more directly and accurately evaluate the swallowing situation in oral, pharyngeal and esophageal stages, and understand the integrity of the protection function of the swallowing airway. It is significance for the diagnosis, selection of intervention methods, and management of Dysphagia in the swallowing stage. VFSS and flexible endoscopic examination of swallowing (FEES) are the gold standard to determine Dysphagia. In addition, pharyngeal cavity pressure measurement is widely carried out in recent years, which can measure the pressure in the pharyngeal cavity and quantify the swallowing function, such as high-resolution pharyngeal cavity pressure measurement (HRM), upper esophageal Sphincter pressure measurement, and automatic pharyngeal impedance pressure measurement.

Compared to VFSS, the above evaluation methods have their unique advantages and unavoidable drawbacks. FEES can observe the transport of food pellets to the throat during swallowing under direct view of the monitor, and observe the deformation and displacement of food pellets in the throat. Therefore, FEES can better reflect the anatomical structure of the throat and the accumulation of food masses compared to VFSS, and is more suitable for swallowing dysfunction caused by cranial neuropathy, postoperative or traumatic injuries, and anatomical structural abnormalities. It is also suitable for research on aspiration (24, 25). Another advantage of FEES is that it has no X-ray radiation and can be repeatedly checked. The device is easy to carry and can be checked by the bedside. However, FEES cannot directly observe the entire process of food mass transportation, and can only judge the swallowing effect through indirect information on the distribution of food mass in the pharynx after swallowing. Without hunger, the opening of the cricopharyngeal muscle can be directly observed. Therefore, it cannot directly evaluate the coordination between swallowing organs. When the swallowing amount reaches its maximum or the food covers the laryngoscope lens, it will not be imaged. HRM can dynamically and continuously reflect the changes in pharyngeal pressure throughout the swallowing process, with a focus on reflecting pharyngeal coordination. The disadvantage is that it is not possible to directly see the anatomical structure and food passage status, nor can it determine whether there is aspiration (26). Meanwhile, VFSS still has certain drawbacks. If it wants to receive X-ray radiation, it needs to be transferred to the radiology department, which cannot reflect the sensory function of the pharynx. The most important thing is that it cannot quantitatively analyze the pharyngeal muscle contraction force and the pressure inside the food mass, and much of the information recorded during VFSS cannot be fully utilized. However, the location can be determined and symptoms of dysphagia can be observed by angiography during swallowing. Previous studies have confirmed that dynamic contrast quantitative analysis technology can effectively clarify the relationship between the movements of the organs involved in swallowing as a food bolus passes (27–29). Applying VFSS to the analysis of muscle relaxation and contraction of the upper sphincter of the esophagus and pharynx can provide more detailed information than can be determined by assessments based on contrast alone. Determining the relationship between quantitative and descriptive findings could be useful in clinical practice, although it was previously unknown whether such outcomes would consistently match up. Therefore, the VFSS qualitative and quantitative results were compared and analyzed in this study.

The quantitative values of the kinematic parameters differed based on the qualitative grades of the VFSS. First, weakening of the movement of the hyoid–laryngeal complex resulted in lower HSM and HAM values, confirming the weaker the movement of the hyoid laryngeal bone complex, the greater the degree of swallowing dysfunction. This finding is consistent with the results of other studies that have investigated swallowing physiology and pathology (17, 30). Movement of the hyoid–laryngeal complex is a crucial component of swallowing function, as it helps ensure the closure of the throat, the return of the epiglottis, the opening of the cricopharyngeal muscle, and the smooth and safe completion of swallowing activities. Measuring the displacement of the hyoid bone is often used to quantify the movement ability of the hyoid–laryngeal complex. During swallowing, the vertical movement of the hyoid bone drives the closure of the epiglottis, which is beneficial for the protection of the airway, whereas the forward movement of the hyoid is beneficial for the opening of the UES (31). Theoretically, upward and forward displacement of the hyoid bone plays a positive role in swallowing. The results of this study also confirmed that the measuring the displacement of the hyoid bone can help to objectively evaluate the motion amplitude of the hyoid-laryngeal complex and can compensate for the lack of information provided in clinical evaluations based solely on observation of tongue extension or swallowing angiography to describe the motion of the hyoid-laryngeal complex.

An increase in the cricopharyngeal muscle opening duration, however, did not significantly alter the opening diameter of the UES. Similarly, the opening duration of the UES did not change significantly with the different qualitative total value categories. Physiologically, the coordination of activities involved in swallowing mainly the coordination of UES relaxation and pharyngeal muscle contraction, as well as the sequential movement of upper and lower pharyngeal muscle contractions. The evaluation of swallowing coordination via VFSS is mainly based on the observation and description of the opening of the cricopharyngeal muscle; however, such observations may be subjective, and no unified diagnostic criteria have been established. In contrast, quantitative analysis allows for the assessment of the opening range of the UES, which is an important quantitative index that reflects the coordination of the swallowing process (32). The quantitative analysis of the VFSS data facilitated the measurement of the opening range of the UES, although no obvious difference with qualitative total value; one possible explanation for this could be related to the primary disease associated with the dysphagia among the patients included in this study. Previous studies have reported that coordination of movements involved in swallowing is regulated by the swallowing pattern generator within the brainstem (33, 34). The development of a brainstem lesion usually manifests as weakening of the pharynx’s ability to push and/or an abnormal UES relaxation function, which can easily lead to serious consequences that include leakage or aspiration. However, the present study did not include patients with such brainstem diseases; therefore, the value of the UES opening diameter does not reliably reflect the relaxation of the cricopharyngeal muscle.

There was no significant difference in the pharyngeal area at rest between the different qualitative grades of vallecular and pyriform fossa residues. The pharyngeal area at rest can be used as another objective index for evaluating the coordination of the pharyngeal phase of swallowing. The pharyngeal cavity contraction rate reflects the degree of contraction during swallowing in the pharyngeal phase (35, 36). In this stage, the hyoid bone on the larynx moves upward, the arytenoepiglottis and thyrohyoid muscles contract, and the base of the tongue inclines backward to ensure the epiglottis forms a proper cover; while the epiglottis valley on both sides is oriented close to the midline, the muscle group in the larynx contracts, the vocal cord and the ventricular band retracts, the glottis closes, and the pharyngeal constrictor retracts. During this time, the laryngopharynx and pyriform fossa are open, and the food mass is squeezed across the epiglottis, reaching the esophageal entrance; the opening of the upper esophageal sphincter is coordinated to ensure smooth passage of food through the pharyngeal cavity for entry into the esophagus. Thus, the main function of the pharyngeal cavity and the related muscle contraction is to clear the pharyngeal mass and squeeze the food bolus downward into the esophagus during swallowing. When swallowing disorders are caused by various organic and neuromuscular abnormalities, the ability of the pharyngeal cavity to clear food decreases, the corresponding size of the food mass remaining in the pharyngeal cavity increases, and the corresponding pharyngeal cavity contraction rate decreases. Aspiration can easily occur when the glottis reopens. Previous studies have shown that in the treatment of dysphagia, improving pharyngeal contraction can effectively reduce the residue remaining after swallowing (37). However, the pharyngeal cavity contraction rate in this study did not significantly correlate with the grading of epiglottic valley and pyriform fossa residues. Considering the limited inclusion of primary diseases in this study, measurements of the pharyngeal cavity area and contraction rate may have had little correlation with the presence of such residues. In addition, the results of this study revealed that 96.58% of the patients had vallecular residues, 83.76% had pyriform fossa residues, and the average contraction rate of the pharyngeal cavity was 40–55%. Therefore, it is also possible that the 5 mL volume of food paste administered in this study was too small, which could have resulted in weak sensory and motor stimulation of the pharynx, thereby affecting the contraction of the pharyngeal constrictor muscle and resulting in insufficient peristalsis.

In this study, most of the quantitative and time parameter values showed statistically significant differences according to the different qualitative grades assigned during the VFSS, including the oral transit time, swallowing reaction time in the pharyngeal phase, soft palate lift duration, hyoid movement duration, pharyngeal cavity transit time, and LVC duration. However, the UES opening duration poorly reflected the degree of cricopharyngeal muscle opening, which is consistent with the UES opening diameter results.

All quantitative values were measured or calculated built-in software tools and formulas, reflecting a portion of the time sequence and interval during the swallowing process. A factor analysis for dimensionality reduction of the 12 quantitative variables was conducted, and the results suggested that the quantitative items were relatively independent. The five principal components selected barely represented all of the quantitative values; that is, the measurements of the 12 quantitative variables could still adequately describe the entire swallowing process. The results of this study also show that there is a low correlation between the quantitative total value and the qualitative total value, which means quantitative results can not reveal that the correlation and sensitivity with qualitative results. However, since the types and definitions of the parameters used by various institutions are not yet unified, this study suggests that in the future selection of quantitative parameters of VFSS, studies should continue to optimize the existing parameters and attempt to screen out and standardize effective and comprehensive parameters to fully describe the swallowing process. This could help promote their use in clinical settings to better evaluate the effects on patients before and after treatment or the differences between patients.

The quantitative of VFSS based on pathological samples collected from a wide range of individuals with multiple diseases, the extraction of the most effective and valuable information from dysphagia angiography and the objective comparison of the levels of functionality within-patients before and after treatment, as well as between patients, can fully meet the comprehensive needs of scientific research, stimulate more innovative research, and generate ideas and references for the evaluation and follow-up treatment of dysphagia. For now, the quantitative analysis of VFSS is mainly used to describe the physiological state of swallowing (14, 38), explore the pathological and physiological characteristics of swallowing in different diseases (23, 39), analyze the effects of age, gender, texture of food balls and other factors on swallowing (40, 41), and evaluate treatment efficacy (42, 43). In the future, the quantitative results should be used for evaluation, and future studies should assess other valuable parameters and improve those with poor reliability, validity, and matching. With progress in science and technology and further deepening of research in this field, fully automated quantitative analysis of VFSS data could become possible, improving the effectiveness of swallowing assessments and reducing the burden on clinical workers.

## 5. Strengths and limitations

This study has the following limitations: (1) the types of patients with dysphagia selected in the study was relatively limited, the patients with PD were in phase 1–2, and the existing dysphagia was relatively mild, and the representativeness of the sample was relatively weak, so further research must be conducted in the future with an improved design; (2) the type of food balls selected was relatively fixed, which could have had a certain impact on the results; (3) the sample size needs to be further expanded; (4) during video acquisition, due to the patients’ conditions, the body position, head control, and degree of cooperation could have been impacted, among other factors, which could have affected the clarity of imaging of various anatomical components, resulting in difficulties and errors in the qualitative analysis; (5) when obtaining various quantitative results, semi-automatic methodologies may lead to some measurement errors due to deviations of the measurer’s understanding of the measurement technique, and the workload of the data acquisition process is large with many steps, so fatigue could have led to some measurement errors; and (6) considering the imaging factors, the radiation amplification effect could give the impression that the distance between two points on the image is larger than the actual value, and the radial distortion of the ray could stretch the length of the structure around the image. Radiation amplification and radial distortion of the rays may have affected the accuracy of the analysis.

## 6. Conclusion

In conclusion, there was a good match between the qualitative and quantitative VFSS time parameter values. However, the kinematic parameters did not accurately reflect the quantitative results. Determining quantitative values can still sufficiently describe the entire swallowing process, and these measures positively correlated with results of the qualitative evaluations. It is recommended that the quantitative evaluation parameters be optimized in future studies to facilitate assessments of swallowing function in patients with dysphagia.

## Data availability statement

The original contributions presented in the study are included in the article/supplementary material, further inquiries can be directed to the corresponding authors.

## Ethics statement

The study was conducted in accordance with the Declaration of Helsinki and was approved by the Ethics Committee of Beijing Rehabilitation Hospital (protocol code 2020-008 and approved on December 1, 2020). Informed consent was obtained from all participants involved in the study. The patients/participants provided their written informed consent to participate in this study. Written informed consent was obtained from the individual(s) for the publication of any potentially identifiable images or data included in this article.

## Author contributions

XD designed this study. QC, BW, WJ, and HW collected the data. QC analyzed the data and drafted the manuscript. YH and QZ interpreted and critically assessed the results. JX and XD further revised the manuscript. All authors contributed to the article and approved the submitted version.

## Funding

This work was funded by the Science and Technology Development Program of the Beijing Rehabilitation Hospital (2020-008) and the Science and Technology Development Program of the Beijing Rehabilitation Hospital (2021-021).

## Conflict of interest

The authors declare that the research was conducted in the absence of any commercial or financial relationships that could be construed as a potential conflict of interest.

## Publisher’s note

All claims expressed in this article are solely those of the authors and do not necessarily represent those of their affiliated organizations, or those of the publisher, the editors and the reviewers. Any product that may be evaluated in this article, or claim that may be made by its manufacturer, is not guaranteed or endorsed by the publisher.

## References

[1] McCarty EB Chao TN. Dysphagia and swallowing disorders. Med Clin N Am. (2021) 105:939–54. doi: 10.1016/j.mcna.2021.05.013 34391544

[2] Banda KJ Chu H Kang XL Liu D Pien LC Jen HJ. Prevalence of dysphagia and risk of pneumonia and mortality in acute stroke patients: a meta-analysis. BMC Geriatr. (2022) 22:420. doi: 10.1186/s12877-022-02960-5 35562660 PMC9103417

[3] Suttrup I Warnecke T. Dysphagia in Parkinson's disease. Dysphagia. (2016) 31:24–32. doi: 10.1007/s00455-015-9671-9 26590572

[4] Wang P Wang B Chen X Xiong B Xie F Wu S. Six-year follow-up of dysphagia in patients with Parkinson's disease. Dysphagia. (2022) 37:1271–8. doi: 10.1007/s00455-021-10387-0, PMID: 34826007

[5] Rommel N Hamdy S. Oropharyngeal dysphagia: manifestations and diagnosis. Nat Rev Gastroenterol Hepatol. (2016) 13:49–59. doi: 10.1038/nrgastro.2015.199 26627547

[6] Panebianco M Marchese-Ragona R Masiero S Restivo DA. Dysphagia in neurological diseases: a literature review. Neurol Sci. (2020) 41:3067–73. doi: 10.1007/s10072-020-04495-2, PMID: 32506360 PMC7567719

[7] Thiyagalingam S Kulinski AE Thorsteinsdottir B Shindelar KL Takahashi PY. Dysphagia in Older Adults. Mayo Clin Proc. (2021) 96:488–97. doi: 10.1016/j.mayocp.2020.08.001 33549267

[8] Turkington L Nund RL Ward EC Farrell A. Exploring current sensory enhancement practices within Videofluoroscopic swallow study (VFSS) clinics. Dysphagia. (2017) 32:225–35. doi: 10.1007/s00455-016-9747-1, PMID: 27586878

[9] Allen J Blair D Miles A. Assessment of videofluoroscopic swallow study findings before and after cricopharyngeal myotomy. Head Neck. (2017) 39:1869–75. doi: 10.1002/hed.24846 28644552

[10] Giraldo-Cadavid LF Leal-Leaño LR Leon-Basantes GA Bastidas AR Garcia R Ovalle S. Accuracy of endoscopic and videofluoroscopic evaluations of swallowing for oropharyngeal dysphagia. Laryngoscope. (2017) 127:2002–10. doi: 10.1002/lary.26419, PMID: 27859291

[11] Kahrilas PJ Lin S Rademaker AW Logemann JA. Impaired deglutitive airway protection: a videofluoroscopic analysis of severity and mechanism. Gastroenterology. (1997) 113:1457–64. doi: 10.1053/gast.1997.v113.pm9352847, PMID: 9352847

[12] Perlman AL VanDaele DJ Otterbacher MS. Quantitative assessment of hyoid bone displacement from video images during swallowing. J Speech Hear Res. (1995) 38:579–85. doi: 10.1044/jshr.3803.579, PMID: 7674650

[13] Lan Y Xu G Dou Z Lin T Yu F Jiang L. The correlation between manometric and videofluoroscopic measurements of the swallowing function in brainstem stroke patients with dysphagia. J Clin Gastroenterol. (2015) 49:24–30. doi: 10.1097/MCG.0000000000000100, PMID: 24583749

[14] Mancopes R Peladeau-Pigeon M Barrett E Guran A Smaoui S Pasqualoto AS. Quantitative Videofluoroscopic analysis of swallowing physiology and function in individuals with chronic obstructive pulmonary disease. J Speech Lang Hear Res. (2020) 63:3643–58. doi: 10.1044/2020_JSLHR-20-00154, PMID: 33105085 PMC8582841

[15] Postuma RB Berg D Stern M Poewe W Olanow CW Oertel W. MDS clinical diagnostic criteria for Parkinson's disease. Mov Disord. (2015) 30:1591–601. doi: 10.1002/mds.26424 26474316

[16] Expert Consensus Group on Rehabilitation Assessment and Treatment of Dysphagia in China. China dysphagia rehabilitation evaluation and treatment expert consensus group China dysphagia evaluation and treatment expert consensus (2017) part I evaluation [J]. Chin J Phys Med Rehabil. (2017) 39:881–92. doi: 10.3760/cma.j.issn.0254-1424.2017.12.001

[17] Kim Y McCullough GH. Maximum hyoid displacement in normal swallowing. Dysphagia. (2008) 23:274–9. doi: 10.1007/s00455-007-9135-y, PMID: 17962998

[18] Wei KC Hsiao MY Wang TG. The kinematic features of hyoid bone movement during swallowing in different disease populations: a narrative review. J Formos Med Assoc. (2022) 121:1892–9. doi: 10.1016/j.jfma.2022.04.007 35469721

[19] Park CH Kim K Hwang JT Choi JH Lee YT Park YS. Comparison of methods for evaluation of upper esophageal sphincter (UES) relaxation duration: Videofluoroscopic swallow study versus high-resolution manometry. Medicine (Baltimore). (2022) 101:e30771. doi: 10.1097/MD.0000000000030771, PMID: 36181078 PMC9524913

[20] Smaoui S Mancopes R Simmons MM Peladeau-Pigeon M Steele CM. The influence of sex, age, and repeated measurement on pixel-based measures of pharyngeal area at rest. J Speech Lang Hear Res. (2023) 66:1–9. doi: 10.1044/2022_JSLHR-22-00465, PMID: 36780312 PMC10205107

[21] Soares TJ Moraes DP de Medeiros GC Sassi FC Zilberstein B De Andrade CR. Oral transit time: a critical review of the literature. Arq Bras Cir Dig. (2015) 28:144–7. doi: 10.1590/S0102-67202015000200015, PMID: 26176255 PMC4737340

[22] Dai M Wan GF Wang YY Wei XM Xie CQ Wu HX. The reliability of qualitative analyses of video fluoroscopic images[J]. Chin J Phys Med Rehabil. (2015) 37:908–12. doi: 10.3760/cma.j.issn.0254-1424.2015.012.005

[23] Waito AA Plowman EK Barbon CEA Peladeau-Pigeon M Tabor-Gray L Magennis K. A cross-sectional, quantitative Videofluoroscopic analysis of swallowing physiology and function in individuals with amyotrophic lateral sclerosis. J Speech Lang Hear Res. (2020) 63:948–62. doi: 10.1044/2020_JSLHR-19-00051, PMID: 32310713 PMC7242989

[24] The European Society for Swallowing Disorders Dziewas R Baijens L Schindler A Verin E Michou E. European Society for Swallowing Disorders FEES accreditation program for neurogenic and geriatric oropharyngeal dysphagia. Dysphagia. (2017) 32:725–33. doi: 10.1007/s00455-017-9828-9, PMID: 28779300 PMC5674114

[25] Dziewas R Glahn J Helfer C Ickenstein G Keller J Ledl C. Flexible endoscopic evaluation of swallowing (FEES) for neurogenic dysphagia: training curriculum of the German Society of Neurology and the German stroke society. BMC Med Educ. (2016) 16:70. doi: 10.1186/s12909-016-0587-3 26911194 PMC4766659

[26] Walczak CC Jones CA McCulloch TM. Pharyngeal pressure and timing during bolus transit. Dysphagia. (2017) 32:104–14. doi: 10.1007/s00455-016-9743-5, PMID: 27565155 PMC5832365

[27] Dharmarathna I Miles A Allen J. Predicting penetration-aspiration through quantitative swallow measures of children: a videofluoroscopic study. Eur Arch Otorhinolaryngol. (2021) 278:1907–16. doi: 10.1007/s00405-021-06629-4 33564910

[28] Dharmarathna I Miles A Fuller L Allen J. Quantitative video-fluoroscopic analysis of swallowing in infants. Int J Pediatr Otorhinolaryngol. (2020) 138:110315. doi: 10.1016/j.ijporl.2020.110315, PMID: 32861978

[29] Matsuo K Palmer JB. Video fluoroscopic techniques for the study of Oral food processing. Curr Opin Food Sci. (2016) 9:1–10. doi: 10.1016/j.cofs.2016.03.004, PMID: 27213138 PMC4871608

[30] Matsuo T Matsuyama M Nakatani K Mori N. Evaluation of swallowing movement using ultrasonography. Radiol Phys Technol. (2020) 13:62–8. doi: 10.1007/s12194-019-00547-1 31786806

[31] Azpeitia Armán J Lorente-Ramos RM Gete García P Collazo Lorduy T. Videofluoroscopic evaluation of Normal and impaired oropharyngeal swallowing. Radiographics. (2019) 39:78–9. doi: 10.1148/rg.2019180070 30620692

[32] Dziewas R Michou E Trapl-Grundschober M Lal A Arsava EM Bath PM. European stroke organisation and European Society for Swallowing Disorders guideline for the diagnosis and treatment of post-stroke dysphagia. Eur Stroke J. (2021) 6:LXXXIX–CXV. doi: 10.1177/23969873211039721, PMID: 34746431 PMC8564153

[33] Ertekin C. Neurogenic dysphagia in brainstem disorders and EMG evaluation. J Basic Clin Health Sci. (2017) 1:1–10. doi: 10.5152/jbachs.2017.96

[34] Huckabee ML Lamvik K Jones R. Pharyngeal mis-sequencing in dysphagia: characteristics, rehabilitative response, and etiological speculation. J Neurol Sci. (2014) 343:153–8. doi: 10.1016/j.jns.2014.05.064, PMID: 24954087

[35] Boaden E Burnell J Hives L Dey P Clegg A Lyons MW. Screening for aspiration risk associated with dysphagia in acute stroke. Cochrane Database Syst Rev. (2021) 10:CD012679. doi: 10.1002/14651858.CD012679 34661279 PMC8521523

[36] Weng W Imaizumi M Murono S Zhu X. Expert-level aspiration and penetration detection during flexible endoscopic evaluation of swallowing with artificial intelligence-assisted diagnosis. Sci Rep. (2022) 12:21689. doi: 10.1038/s41598-022-25618-z, PMID: 36522385 PMC9753025

[37] Stokely SL Peladeau-Pigeon M Leigh C Molfenter SM Steele CM. The relationship between pharyngeal constriction and post-swallow residue. Dysphagia. (2015) 30:349–56. doi: 10.1007/s00455-015-9606-5, PMID: 25920993 PMC4469308

[38] Ambrocio KR Miles A Bhutada AM Choi D Garand KL. Defining Normal sequential swallowing biomechanics. Dysphagia. (2023). doi: 10.1007/s00455-023-10576-z, PMID: 37097448 PMC11554329

[39] Stevens M Mayerl CJ Bond L German RZ Barkmeier-Kraemer JM. Pathophysiology of aspiration in a unilateral SLN lesion model using quantitative analysis of VFSS. Int J Pediatr Otorhinolaryngol. (2021) 140:110518. doi: 10.1016/j.ijporl.2020.110518, PMID: 33310447 PMC7770015

[40] Kang BS Oh BM Kim IS Chung SG Kim SJ Han TR. Influence of aging on movement of the hyoid bone and epiglottis during normal swallowing: a motion analysis. Gerontology. (2010) 56:474–82. doi: 10.1159/000274517 20068282

[41] Stokely SL Molfenter SM Steele CM. Effects of barium concentration on oropharyngeal swallow timing measures. Dysphagia. (2014) 29:78–82. doi: 10.1007/s00455-013-9485-6 24045851 PMC3921461

[42] Liu HC Williamson CW Zou J Todd JR Nelson TJ Hill LM. Quantitative prediction of aspiration risk in head and neck cancer patients treated with radiation therapy. Oral Oncol. (2023) 136:106247. doi: 10.1016/j.oraloncology.2022.106247, PMID: 36410204

[43] Wang J Yang C Wei X Zhang M Dai M Huang G. Videofluoroscopic swallowing study features and resting-state functional MRI brain activity for assessing swallowing differences in patients with mild cognitive impairment and risk of dysphagia. Dysphagia. (2023) 38:236–46. doi: 10.1007/s00455-022-10460-2, PMID: 35556171

